# Infra-tentorial brain tumor subtypes in children and adults—surgical outcome in an ethnic population with a single regional tertiary center

**DOI:** 10.1186/s41016-022-00275-3

**Published:** 2022-05-03

**Authors:** Abdul Rashid Bhat, Muhammed Afzal Wani, Altaf Rehman Kirmani

**Affiliations:** Department of Neurosurgery, Sher-i-Kashmir Institute of Medical Sciences (SKIMS), Srinagar, Kashmir India

**Keywords:** Infra-tentorial brain tumors, Histological subtypes, Ethnic population, Surgical outcome

## Abstract

**Background:**

To analyze clinically and radiologically the surgical outcome like residual disease, progression of disease, recurrence, disabilities, event-free survival (EFS), and mortality of different infra-tentorial tumor subtypes in children and adults of a strictly non-migratory and ethnic population.

**Methods:**

The 410 histologically proved, out of 589, infra-tentorial brain tumor patients were analyzed clinically and by the imaging post-surgically in a single tertiary center for an ethnic region. In this analytico-observational study, retrospectively postoperative records of 589 infra-tentorial brain tumors from November 1998 to December 2018 (20 years) were retrieved, scrutinized, and compiled. The post-operative clinic-radiological records of 410 patients with proved histopathological examination results were included. Statistical law of variance was applied where-ever necessary.

**Results:**

The 63.2% of the all 410 operated infra-tentorial brain tumors were males while females predominated in meningiomas and pineoblastomas. About 31.7% infra-tentorial tumors were children (below 18 years). About 54.1% cases were histologically malignant. The residual tumors comprised 40.2% and symptoms of disease-progression occurred in 10.9%. The tumor recurrence occurred in 14.3% while 6.0% patients developed severe disability. The overall mortality was 11.4% but 18.9% in malignant tumors. The event-free survival (EFS) for all the patients was 66.0%, patients with malignancies had 47.7% and benign group had 87.7%.

**Conclusion:**

The study, surgical outcome of infra-tentorial brain tumor subtypes in children and adults (approx. 1/3rd of patients being children), conducted in a tertiary center at a remote land-locked location with non-migratory ethnic population as its catchment area, has a significant epidemiological value for the community and the region.

## Background

Anatomically tight-spaced infra-tentorial (posterior fossa) intra-cranial-space of brain, a biologically active tumor subtype and the obstructive hydrocephalus are the three predictors of the worse surgical outcome in the infra-tentorial space brain tumors. The infra-tentorial space of the cranial cavity, limited by the tentorium above, also called posterior fossa, has much smaller volume than the rest of the cranial cavity. However the contents of such a comparably small space are several types of motor and sensory tracts and a number of vital nuclei and reticular formation for the systemic body functions and consciousness in the form of midbrain, pons and medulla. Also, packed are cranial nerves, vascular network with large venous sinuses, changing volume of CSF in the ventricle and cisterns and prominently visible cerebellar parenchyma with nuclei and peduncles. Most of the times, the infra-tentorial space tumors present themselves as an acute emergencies following the compression of the brainstem either due to the increase in tumor size, edema, bleed, or its obstruction to CSF pathways and finally herniation. The surgical debulking, to relieve the CSF obstruction and the pressure on the brainstem, though full of risks, is indispensable mode of management. However, given such a small space, the intra-operative complications and post-surgical disease progression owing to residual or recurrence of the lesion worsens the surgical outcome. In 1930, an account of 61 patients of infra-tentorial space tumors of brain was published by the most cherished neurosurgeon of the world, Cushing H, claiming fatal outcome in almost all [1]. The present study outlines the surgical outcome clinico-radiologically in different histological subtypes of infra-tentorial brain tumors in adults and children in a single and regionally ethnic tertiary center.

## Methods

The patients of this study are mainly an ethnic and strictly non-migratory population who belong to a remote land locked snow bound region catered by a single tertiary center with a neurosurgical unit. This observational study took into account the records of those patients who were already treated and did not need to identify themselves to the researchers. Since the study was mainly compilation of surgical and histopathological records wherein neither IRB/Ethical approval nor patient consent was required, it provided an epidemiological data about the disease of a particular regional population. The study will benefit the medical and community health census directly. It was conducted on all operated patients of infra-tentorial tumors of brain admitted from November 1998 to December 2018 (20 years) in the division of Neurosurgery where the neurosurgical patients are managed with a standard and uniform protocol. Retrospectively records of all the 589 patients of infra-tentorial space tumors were retrieved from the files in the Medical Records Department, Operation Theatre Register, Out-patient Department files, referral clinics and follow-up files of the supportive departments like medical and radiation oncology, and pathology of this tertiary healthcare facility. However, all the non-operated and histologically undiagnosed patients of infra-tentorial tumors, who were managed by the non-surgical departments like medical oncology, radiation oncology, and neurology for not being amenable to the surgical procedures, were excluded from this study. Though a statistical bias, by excluding these patients, may exist but the number of such patients has been very small. The information about the patient’s bio-data, history, examination, basic routine biochemical, and hematological investigations, all the imaging (CT, MRI), surgical-procedures, intra-operative (frozen/crush) histopathological reports, final histopathological examination reports, postoperative follow-up notes and imaging records (CT, MRI) of only 410 patients were available and were included and recorded. The histopathological and postoperative records of a total of 179 patients were not available due to damage or lost over the years. The available data was analyzed, compiled and conclusions drawn. The statistical law of variance was applied where ever necessary.

## Results

**Fig. 1:**
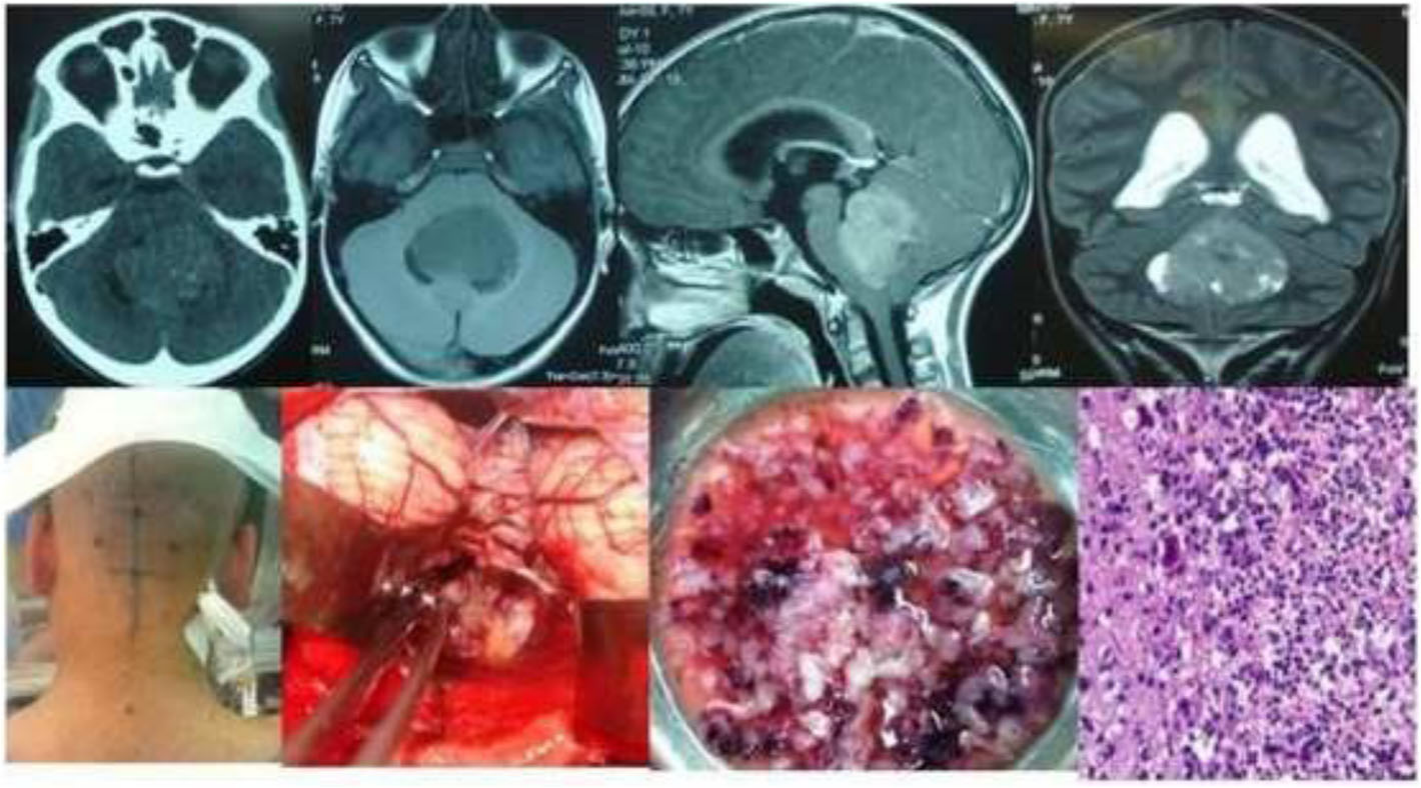
CT scan and MR images of Medulloblastoma in a 12-year-old male child showing intra-operative images in sitting position. Microphotograph (H&E; × 200)

**Fig. 2:**
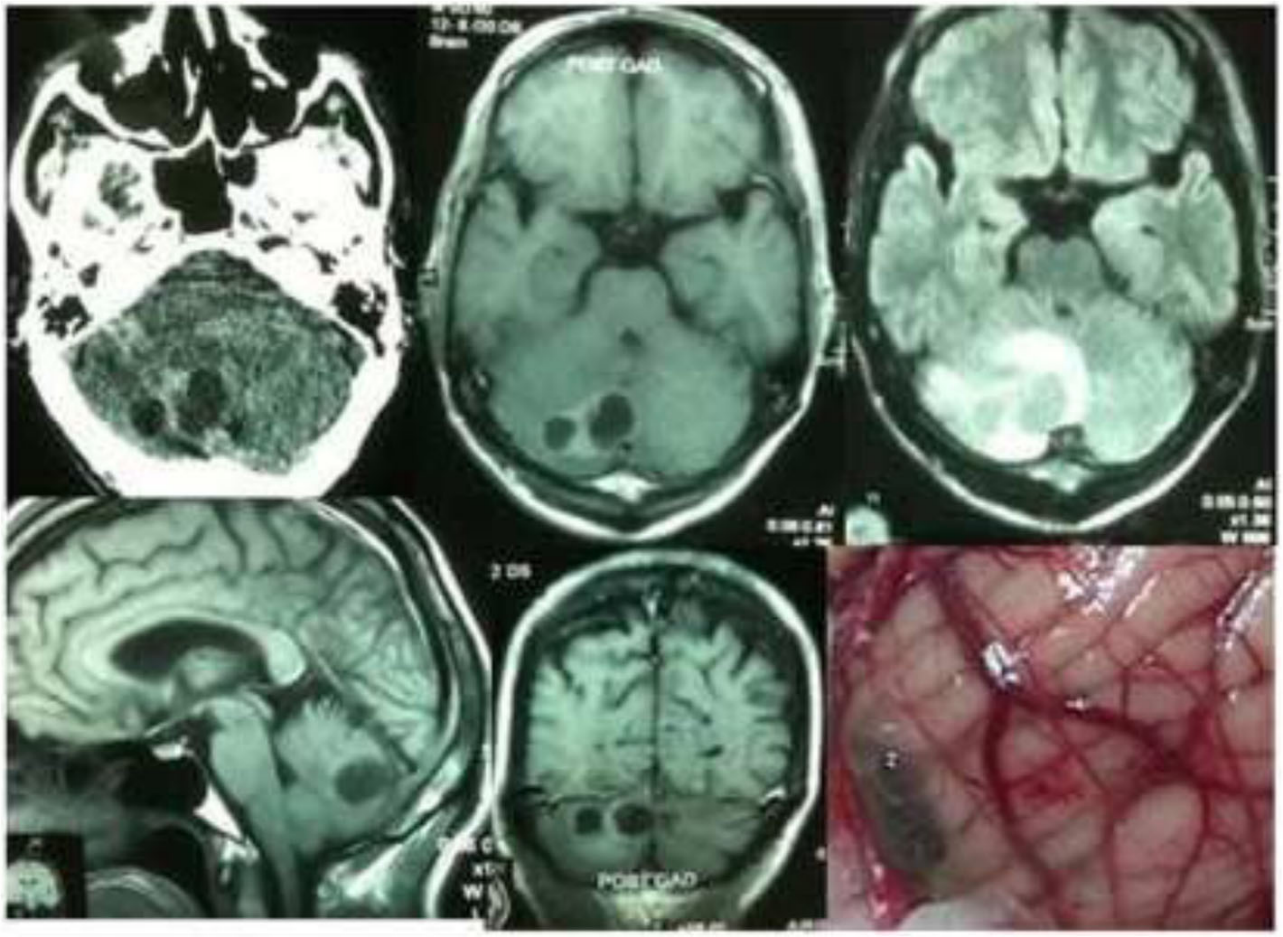
Metastatic lesion in right cerebellar lobe of a 62-year-old female revealed on CT, MR, and intra-operative images

**Fig. 3:**
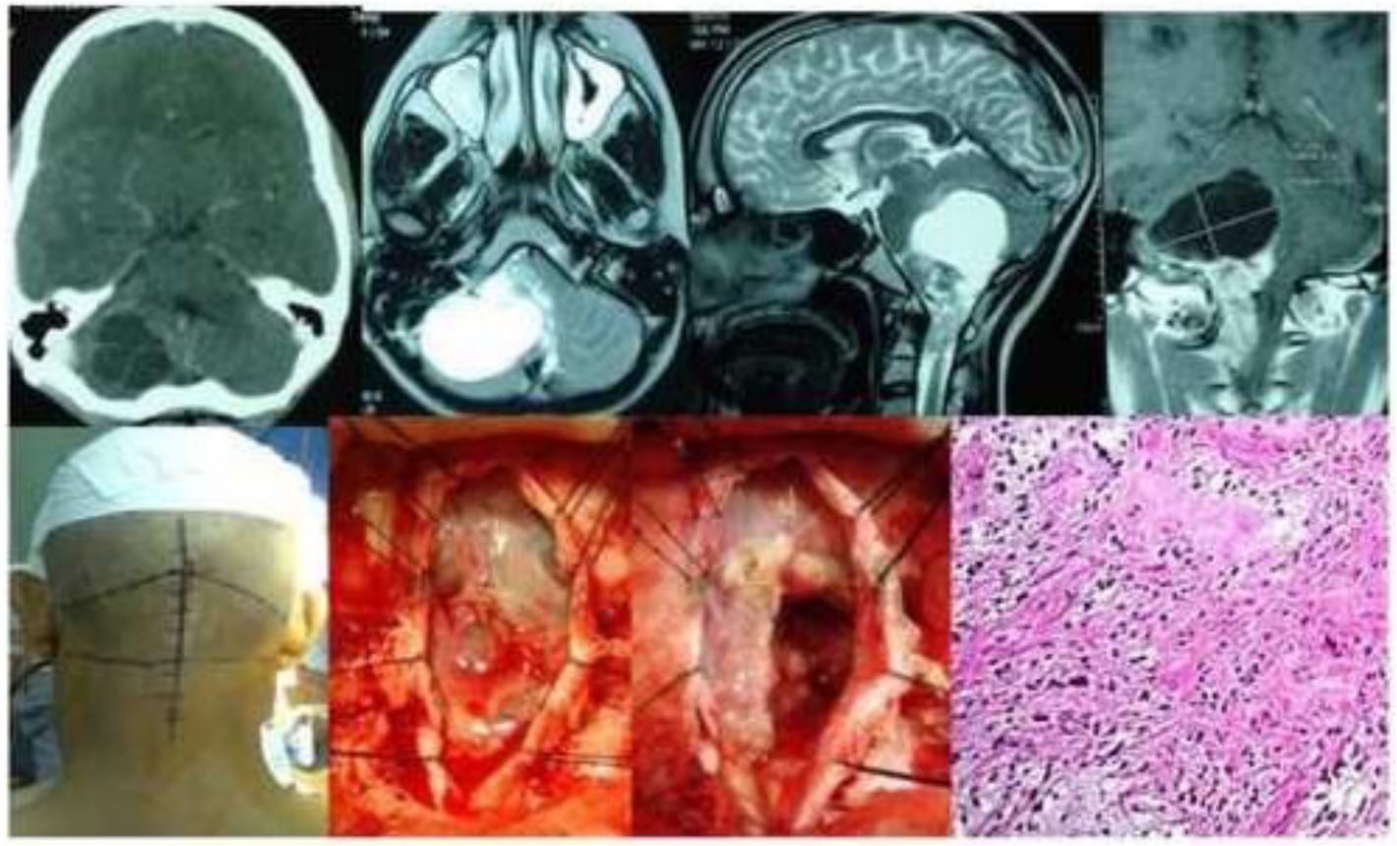
Pilocytic astrocytoma cerebellum of a 22-year-old male is depicted by the CT scan, MR images and intra-operative photographs in sitting position. The histological microphotograph shows cerebellar pilocytic astrocytoma (hematoxylin and eosin × 200)

**Fig. 4:**
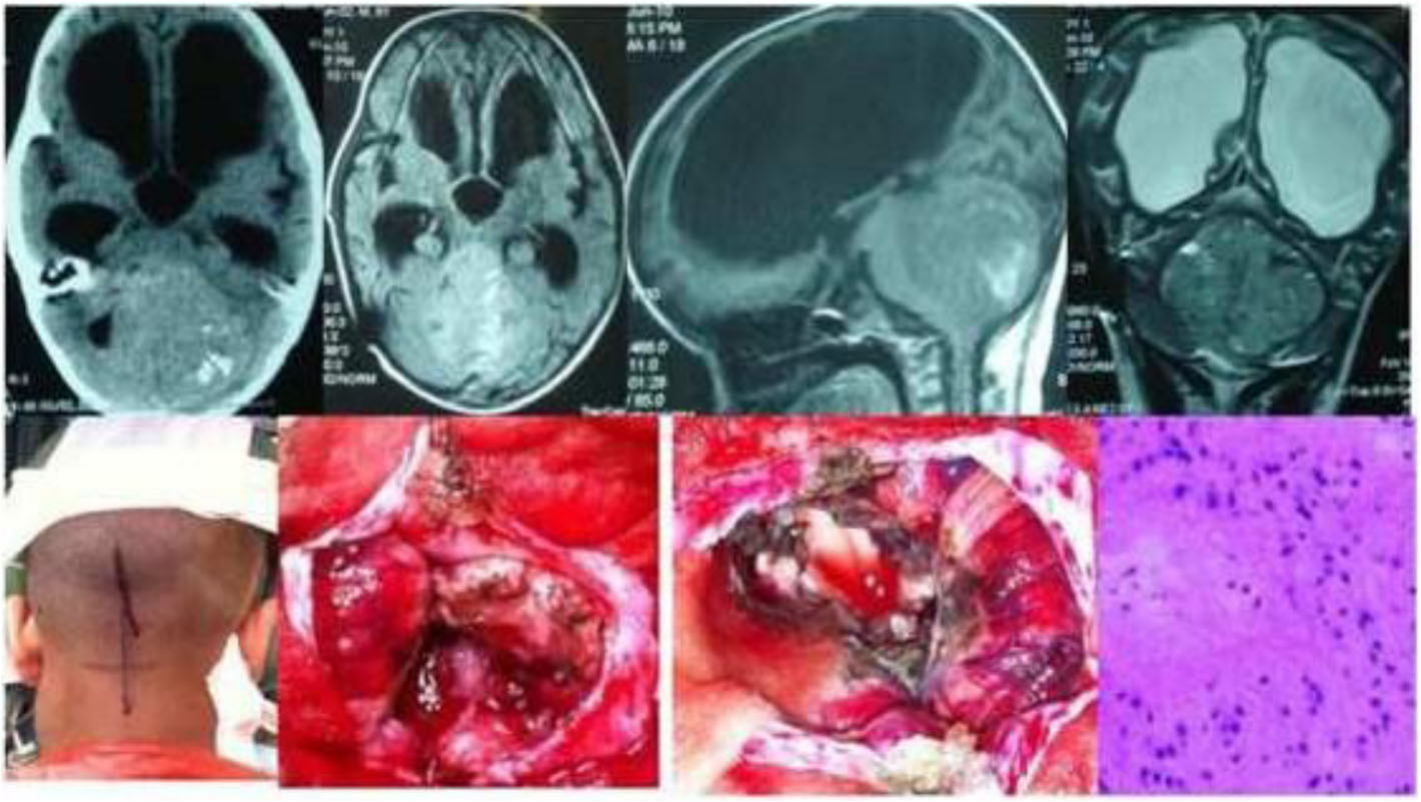
CT, MR, and intra-operative images (sitting position) of a 17-year-old male child with 4th ventricular ependymoma. CT scan depicts calcifications in the lesion. Histological micrograph shows WHO grade II tumor (H&E; × 400)

**Fig. 5:**
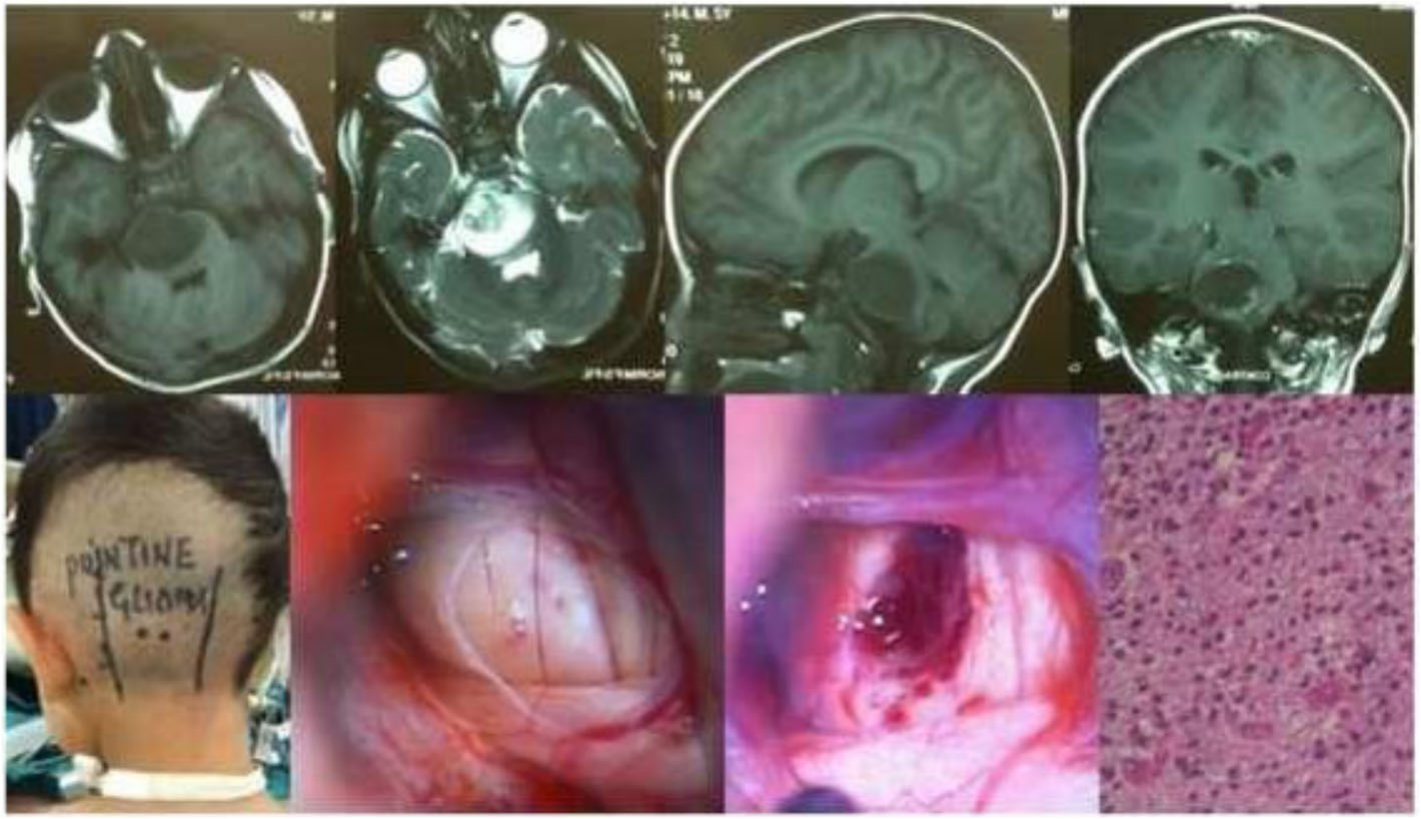
Pontine-glioma grade III in a 5-year-old male child. MR images and intra-operative micro-photographs and clinical photo in sitting position display left retro-mastoid approach and tumor decompression. Histological micrograph (H&E Stain; × 400) is seen

**Fig. 6:**
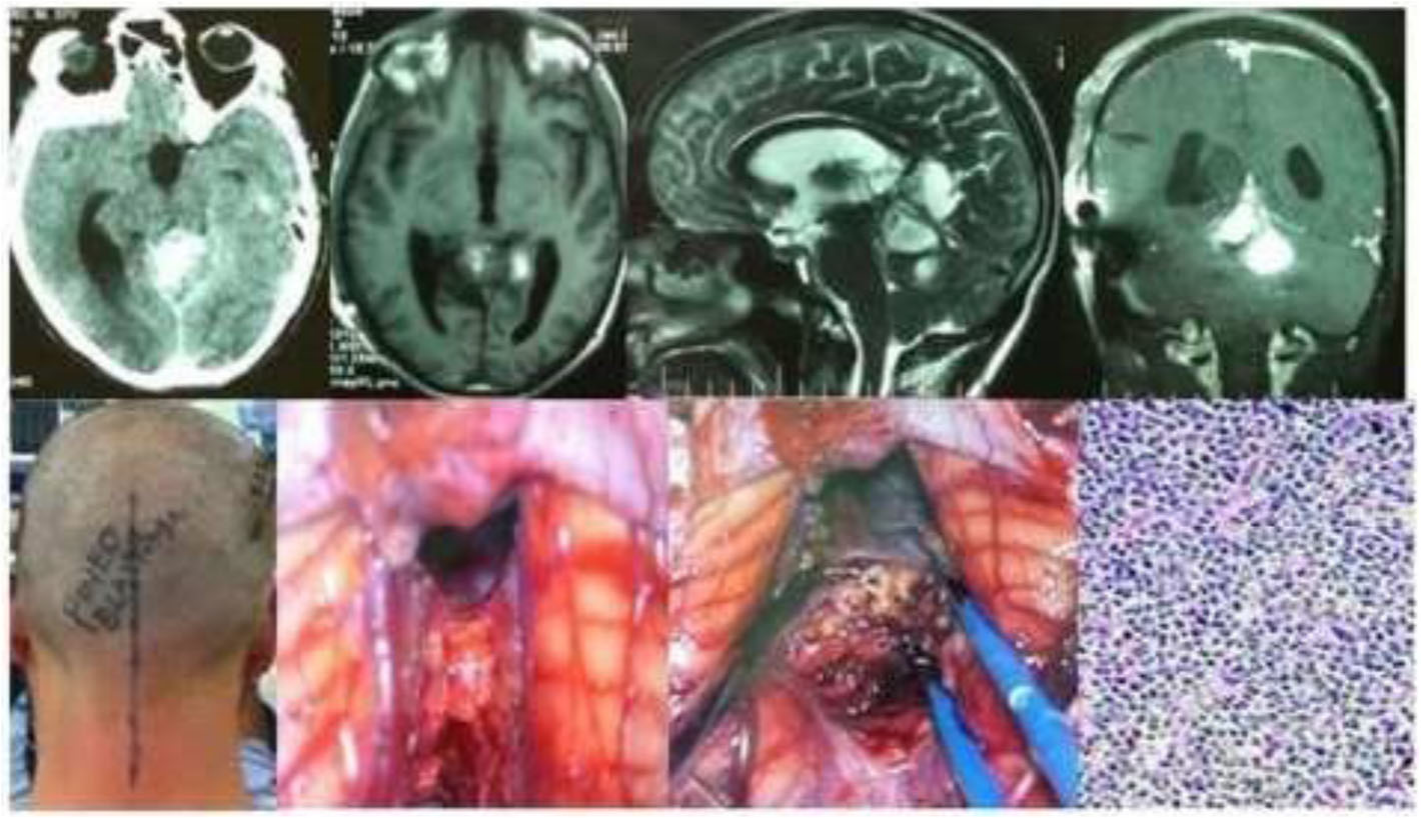
A case of a 16-year-old female pineoblastoma showing CT scan, MR images, and intra-operative photos in sitting position. Photomicrograph reveals pineoblastoma cells on a fibrillar network (H&E Stain; × 300)

Results of the study revealed a male-predominance of 63.2% (259/410) cases in overall infra-tentorial brain tumors with M/F-ratio of 1.7:1.0 (Table 1). About 31.7% (130/410) of all the infra-tentorial tumors were found in the children (age = 18 years and below). The most commonly occurring infra-tentorial tumor subtypes in children were medulloblastomas (Fig. 1), more of a classical variety, 84.7% (61/72). However, benign tumors like schwannomas 2.9% (3/102) and meningiomas 2.6% (1/38) were uncommon while metastases absent in children. The results revealed that the vestibular schwannoma at 23.9% (98/410) was the most occurring individual infra-tentorial space tumor. The most common histological type was the malignancy occurring in 54.1% (222/410) cases. The medulloblastoma (histologically classical) was the commonest (32.4% (72/222)) malignant infra-tentorial tumor. The histologically malignant subtypes of infra-tentorial tumors and the surgical outcome showed significant relation in mortality and morbidity (Tables 1 and 2). The post-surgical imaging in histologically benign tumors showed that 18.6% (35/188) patients had residual disease. The symptoms of disease progression were found in 5.1% (5/98) patients of vestibular schwannomas and 14.2% (4/28) hemangioblastomas. The event-free survival (EFS) of hemangioblastomas was 85.7% (24/28); dermoids 100% (15/15) and it was 88.8% (8/9) for the patients of epidermoids. However, in the infra-tentorial malignant tumors, the postoperative residual tumor on imaging was 70.2% (33/47) in patients of high-grade astrocytomas; 66.6% (6/9) in metastatic lesions (Fig. 2); 62.5% (15/24) pilocytic astrocytomas (Fig. 3); 43.0% (31/72) patients with medulloblastomas and 41.8% (18/43) ependymomas (Fig. 4). The highest tumor recurrences were noted in 100% (4/4) malignant meningiomas (anaplastic and rhabdoid variants); 56.2% (9/16) in brainstem gliomas (Fig. 5); 55.5% (5/9) in metastatic lesions; 42.8% (3/7) in pineoblastomas (Fig. 6) and 19.4% (14/72) in medulloblastomas. The severe neurological deficits and disabilities along with decubitus ulcers and respiratory related infections were more often seen in the brainstem gliomas owing to their slow growth and long survival. No EFS was noted in any case of malignant meningioma, which simultaneously had the highest mortality of 75.0% (3/4). The mortality in metastatic lesions was 66.6% (6/9); brainstem gliomas 43.7% (7/16) and medulloblastomas had 30.5% (22/72) mortality. There was no mortality found in pinealoblastomas and pilocytic astrocytomas, but the high-grade astrocytomas and the ependymomas had a lower mortality of 2.1% (1/47) and 6.9% (3/43) respectively.

**Table 1:** Surgical outcome related to sex and age in infra-tentorial brain tumor subtypes

S. no.	Histopathological type/site	No. of patients	Males	Females	Children(18 years and below)	Postop. clinico-radiological status		
						Residual lesion/dis. Prog.	Recurrence	Mortality
**1.**	**Schwannomas**	**102**	**63**	**39**	**03**	**25/06**	**12**	**04**
	*i). Vestibular (CP angle)*	*98*	60	38	03	23/05	11	04
	*ii). Trigeminal*	*04*	03	01	–	02/01	01	–
**2.**	**Meningiomas:**	38*	**16**	**22**	**01**	**10/03**	**05**	**03**
	*i). Cerebellar cortex and tent.*	*16*	05	11	01	–	–	–
	*ii). Cerebello-pontine angle*	*12*	05	07	–	05	–	–
	*iii). Foramen magnum*	*07*	03	04	–	04/02	04	02
	*iv). Peri-torcular*	*02*	02	–	–	–	–	–
	*v). Jugular foramen*	*01*	01	–	–	01/01	01	01
**3.**	**Hemangioblastomas**	**28**	**15**	**13**	**01**	**04/04**	**04**	**–**
**4.**	**Dermoids**	**15**	**09**	**06**	**04**	–	–	–
**5.**	**Epidermoids**	**09**	**06**	**03**	**03**	–	–	**01**
**6.**	**Medulloblastomas**	**72**	**53**	**19**	**61**	**31/12**	**14**	**22**
	*i).Classical*	*53*	42	11	51	19/07	07	15
	*ii). Desmoplastic variant*	*11*	08	03	04	07/01	01	01
	*iii). Anaplastic changes*	*05*	03	02	04	03/03	05	05
	*iv). Glial differentiation*	*03*	01	02	02	02/01	01	01
**7.**	**C. Astrocytomas (HG)**	**47**	**32**	**15**	**12**	**33/01**	**02**	**01**
**8.**	**Ependymomas**	**43**	**29**	**14**	**09**	**18/05**	**05**	**03**
**9.**	**C. Pilocytic astrocyto.**	**24**	**18**	**06**	**20**	**15**	**–**	**–**
**10.**	**Brainstem gliomas:**	**16**	**11**	**05**	**12**	**16/06**	**09**	**07**
	*i). J. Pilocytic asstrocytoma*	*05*	03	02	05	05/01	02	01
	*ii). Glioblstoma multiforme*	*04*	03	01	01	04/03	04	04
	*iii). Fibrillary astrocytomas*	*03*	01	02	03	03/01	01	–
	*iv). Gangliogliomas*	*02*	02	–	02	02	–	–
	*v). Oligodendrogliomas*	*01*	01	–	01	01	01	01
	*vi). Primitive neuroectodermal tumor*	*01*	01	–	–	01/01	01	01
**11.**	**Metastatic**							
	**Posterior fossa**	**09**	**05**	**04**	**–**	**06/05**	**05**	**06**
	*i). Carcinoma lung*	*03*	03	–	**–**	02/02	02	03
	*ii). Carcinoma breast*	*03*	–	03	**–**	01/01	01	02
	*iii). Renal cell carcinoma*	*02*	01	01	**–**	02/01	01	–
	*iv). Malignant melanoma*	*01*	01	–		01/01	01	01
**12.**	**Pineoblastomas**	**07**	**02**	**05**	**04**	**07/03**	**03**	**–**
**Total**	**Posterior fossa tumors**	**410** (100%)	**259** (63.2%)	**151** (36.8%)	**130** (31.7%)	**165**(40.2%) **/45**(10.9%)	**59** (14.3%)	**47** (11.4%)

38* 4 out of 38 meningiomas were malignant (WHO grade III), *Postop.* postoperative, *HG* high grade (III-anaplastic and IV-glioblastoma multiforme), *C* cerebellar, astrocyto astrocytomas, *J.* juvenile, *Tent.* tentorial, *Dis. Prog.* disease progression, *CP* cerebellopontine

**Table 2:** Surgical outcome related to histopathological subtypes of infra-tentorial space brain tumors

S. no.	Histological types	No. of patients	Post-surgical clinical/radiological outcome					
			Residual lesion	Symptoms of disease progression	Recurr.	Sev. dis.	EFS	Mort.
**Benign lesions**		**188**	**35**	**10**	**17**	**09**	**165**	**05**
1.	Vestibular (CP angle) schwannomas	98	23	05	11	06	85	04
2.	Trigeminal Schwannomas	04	02	01	01	0	03	0
3.	Meningiomas (grade I, II )	34	06	0	01	03	30	0
	*(i) Meningotheliomatous*	*18*	*0*	*0*	*0*	*01*	*17*	*0*
	*(ii) Fibrous*	*6*	*1*	*0*	*0*	*0*	*06*	*0*
	*(iii) Ttransitional*	*3*	*1*	*0*	*0*	*01*	*02*	*0*
	*(iv) Psammomatous*	*2*	*0*	*0*	*0*	*0*	*02*	*0*
	*(v) Atypical*	*2*	*2*	*0*	*01*	*01*	*00*	*0*
	*(vi) Angiomatous*	*1*	*1*	*0*	*0*	*0*	*01*	*0*
	*(vii) Secretory*	*1*	*1*	*0*	*0*	*0*	*01*	*0*
	*(viii) Microcystic*	*1*	*0*	*0*	*0*	*0*	*01*	*0*
4.	Hemangioblastomas	28	04	04	04	0	24	0
5.	Dermoids	15	0	0	0	0	15	0
6.	Epidermoids	09	0	0	0	0	08	01
**Malignant lesions**		**222**	**130**	**35**	**42**	**16**	**106**	**42**
1.	Medulloblastomas	72	31	12	14	07	40	22
2.	C. astrocytomas (HG)	47	33	01	02	02	15	01
3.	Ependymomas	43	18	05	05	04	22	03
4.	C. pilocytic astrocyto.	24	15	0	0	0	18	0
5.	Brainstem gliomas	16	16	06	09	03	05	07
6.	Metastatic lesions	09	06	05	05	0	02	06
7.	Pineoblastomas	07	07	03	03	0	04	0
8.	Meningiomas (grade III)	04	04	03	04	0	0	03
	*(i)Anaplastic*	*03*	*03*	*03*	*03*	*0*	*0*	*03*
	*(ii)Rhabdoid*	*01*	*01*	*0*	*01*	*0*	*0*	*0*
**Total**	**Posterior fossa lesion**	**410**	**165**	**45**	**59**	**25**	**271**	**47**

*Recurr.* recurrence, *Sev. Dis.* severe disability, *EFS* event-free survival, *Mort.* mortality, *CP* cerebello-pontine, *C* cerebellar, *Astrocyto* astrocytomas, *HG* high grade (III-anaplastic and IV-glioblastoma), *Grade I, II, III* WHO grades

## Discussion

The present study on the surgical outcome of subtypes of infra-tentorial space brain tumors in children and adults observed that about 1/3rd (31.7%) patients were children (18 years. and below). Histopathologically, malignancy featured in most, (54.1%), of the patients while benign tumors occurred in 45.8% patients (Tables 1 and 2). A research study in 1997 revealed that out of the 1000 vestibular schwannoma tumors operated in 962 patients, 2.1% patients had residual tumors; 1.1% patients had severe neurological disability; 5.5% patients had caudal cranial nerve palsies, and 1.1% had mortality [2]. Seol et al. in 2006 analyzed 116 patients of vestibular schwannomas where residual tumor was seen in 77.5% and the recurrence in 17.2%. The gross total resection was the best approach to avoid the recurrence [3]. Yamakami et al. in 2004 revealed 14% residual tumor, 4% neurodeficit, and no mortality in 50 operated patients of vestibular schwannomas [4]. The present study observed 50% residual tumors in trigeminal schwannomas and 23.4% in vestibular schwannomas. About 2.9% schwannomas were found in children. Roberti et al. 2001, wrote that a hundred and sixty one patients of infra-tentorial space meningiomas were operated over a period of 9 years with residual tumors found in 43% patients; progression of disease and recurrence in 13.7% and mortality was found in 2.5% patients [5]. The researchers in 2012 showed postoperative results of 64 patients of infra-tentorial space meningiomas, where recurrence occurred in 15.6% patients, severe neurological deficits in 33%, hydrocephalus in 43.75% patients, and mortality in 3.2% [6]. Hakuba et al. reported 17% mortality and severe neurological deficits in 83% patients in radical excision of clival meningiomas of infra-tentorial space [7]. Couldwell et al. studied 40 males and 69 females, a male female ratio of 1:1.7, with infra-tentorial space (petro-clival) meningiomas postoperatively in which gross total excision was achieved in 69% of the patients, 13% had recurrence or progression of disease [8]. Louis et al. reported a 5-year progression-free survival of approximately 50% [9]. The present analysis of meningiomas showed almost similar results (Tables 1 and 2). Hemangioblastomas are uncommon, highly vascular, well-circumscibed, less than 3% of all CNS tumors and mostly (7.5%) in adult cerebellum and brainstem [10]. The present study found an incidence of 6.8% for hemangioblastomas, including two sisters in a family, with an event-free survival (EFS) of 85.7%. Dermoid cysts represent a rare clinical entity that accounts for 0.1–0.7% of all brain tumors [11]. This study observed that the dermoids comprised 3.6% of all the infra-tentorial space tumors of the brain. The EFS of dermoids was 100%. Epidermoids, also known as cholesteatomas, are pearly tumors and account for approximately 0.1% of all intra-cranial tumors growing by the desquamation of the cyst wall and accumulation of keratin and cholesterol [12]. Zakrzewski et al, studied 216 children with infra-tentorial space tumors below 18th year of age, which depicted male/female ratio of 1.35:1.00. The commonest tumor subtype was pilocytic astrocytoma in 41.5% patients; medulloblastoma in 34.5%; ependymomas 13%, and mixed neuronal-glial tumors in 5.5% patients [13]. Muzmdar D et al. in 2011, while presenting 154 patients (age < 18 years) of Medulloblastoma noted that 92.2% (142 cases) had classical medulloblastomas and 5.1% (8 cases) had desmoplastic variant. The 5-year and 10-year progression-free survival rate was 73% and 41% respectively for average risk disease while for high risk disease, it was 34% [14]. Rutka 1997 noted that medulloblastomas are intra-cranial childhood neoplasm, accounting for 25% of all childhood tumors [15]. Also, Bloom and Bessell in 1990 showed that medulloblastomas in adults account for < 1% of all adult brain tumors [16]. Chan et al. in 2000 found in a study that the recurrence rate for medulloblastomas in adults is approximately 50% to 60%. The median time-to-tumor progression (TTP) and recurrence is approximately 30 months after treatment [17]. In the present study medulloblastomas (Figs. 4 and 5) were found in 17.5% patients, mostly (84.7%) in children. The postoperative residual tumor was found in 43.0% and recurrence in 19.4%. There occurred a mortality of 30.5%. Djalilian and Hall (1998) reported that 53% patients in a study had grade IV malignant cerebellar gliomas and 47% anaplastic (grade III) astrocytomas [18]. The present study observed that 11.4% infra-tentorial brain tumors had high-grade anaplastic and glioblastoma type of malignant cerebellar astrocytomas. The postoperative residual tumor was found in 70.2% and a EFS of 31.9% was observed with a mortality of 2.1%. Witt et al. 2011 reported that the posterior fossa ependymomas comprise two distinct molecular entities, ependymoma posterior fossa A (EPN PFA) and ependymoma posterior fossa B (EPN PFB), with differentiable gene expression profiles [19]. In the present study, ependymoma of the 4th ventricle (Fig. 4) had residual tumors in 41.8% and recurrence in 11.6%. The EFS of 51.1% and a mortality of 6.9% were observed. Desai et al. 2001, reported that the pilocytic cerebellar astrocytomas comprise 25% of all infra-tentorial brain tumors in children [20]. Follow-up of 104 children with cerebellar juvenile pilocytic astrocytomas over a mean period of 8.3 years, Daszkiewicz et al. 2009, found that 57.6% (60/104) patients had permanent neurological deficits while 47 had significant behavioral disorders [21]. A study by Lesniak et al. 2003 observed that among 57 patients of brainstem gliomas, 29 had a total surgical resection, 8 a near total resection (> 90% resection), 15 a subtotal resection (50–90% resection), and 5 a partial resection (< 50% resection). The progression-free survival of all patients was 71.9% at 3 years and 45.6% at 5 years [22]. Donalson et al. 2006 reported high rate of recurrence or progression which often follows an inexorable course of progression, despite chemo/radio therapy [23]. All brainstem gliomas in present study had postoperative residual lesions and 37.5% had progression of disease and 56.2% had recurrence. The severe disability in the brainstem gliomas, like pontine gliomas (Fig. 5), in present study, was more often linked to the long survival, motor dysfunction, decubitus ulcers, and respiratory system infections caused by the early involvement of lower cranial nerves and the long tracts by these low-grade tumors subtypes. The brain stem gliomas had a mortality of 43.7% and an EFS of 31.2%. Sunderland et al. 2016 reported that overall 80% patients underwent gross total resection (GTR), 14 % subtotal resection (STR) and 6 % underwent biopsy of metastatic deposits of infra-tentorial space contents. The median overall survival (OS) was 6.00 months. The 28 day mortality was 7.6 % (*n* = 7) with a peri-operative morbidity of 22.8 % (*n* = 21) [24]. Zhang et al. 2012 observed that the most common primary site of malignancy for brain metastatic deposit was lung (20–40 %) followed by breast (5–17 %) and melanoma (7–11 %) with renal, colorectal, and gynecological cancers making up the majority of the remaining [25]. The present series of 410 infra-tentorial brain tumors consisted of 2.1% patients of metastatic deposits (Fig. 2), mostly from primaries like carcinoma lung, breast, renal cell carcinoma and malignant melanoma. Tate et al. in 2012 suggested an increase in survival of pineoblastomas with increasing degrees of resection by observing 5-year survival rate of 84% for patients who underwent gross total resection versus 53% for patients who underwent subtotal resection and 29% for patients who underwent debulking [26]. Pineoblastomas (Fig. 6) in this study comprised 1.7% of all infra-tentorial brain tumors while 57.1% of these were children. Roberti et al. 2001 reported 5% malignant meningiomas in a study of 161 patients [5]. However, Wang et al. 2016 reported that about 51% patients experienced recurrences. The relapse-free survival at 12 months was 84.3% and at 5 years was 57.8% [27]. Of 410 infra-tentorial space brain tumors presently, 0.97% had malignant meningiomas (WHO grade III), mostly rhabdoid and anaplastic, which formed 10.6% of all infra-tentorial tumors with a recurrence of 100% and mortality of 75.0%.

## Conclusion

The present study, surgical outcome of infra-tentorial space brain tumor subtypes in children (approx. 1/3rd of patients being children) and adults, was conducted in a tertiary center at a remote land-locked location with non-migratory ethnic population as its patient-catchment area and has a significant epidemiological value for the community and the region. Given the aggressive biological behavior, the histologically proven malignant subtypes of the infra-tentorial space have all the potential to harm the vitality of infra-tentorial neural structures and lead to catastrophic outcome pre, intra-, and postoperatively.

## Data Availability

Data sharing not applicable to this article as no datasets were generated or analyzed during the current study.
